# The Roles of Mosquitoes in the Circulation of Monoxenous Trypanosomatids in Temperate Climates

**DOI:** 10.3390/pathogens11111326

**Published:** 2022-11-11

**Authors:** Alexei Y. Kostygov, Marina N. Malysheva, Anna I. Ganyukova, Alexey V. Razygraev, Daria O. Drachko, Vyacheslav Yurchenko, Vera V. Agasoi, Alexander O. Frolov

**Affiliations:** 1 Zoological Institute of the Russian Academy of Sciences, 199034 St. Petersburg, Russia; 2 Life Science Research Centre, Faculty of Science, University of Ostrava, 71000 Ostrava, Czech Republic

**Keywords:** Trypanosomatidae, prevalence, specificity, transmission, *Paratrypanosoma*, *Crithidia*, overwintering mosquitoes, experimental infection, facultative host

## Abstract

Monoxenous (insect-restricted) trypanosomatids are highly diverse and abundant in nature. While many papers focus on the taxonomy and distribution of these parasites, studies on their biology are still scarce. In particular, this concerns trypanosomatids inhabiting the ubiquitous mosquitoes. To shed light on the circulation of monoxenous trypanosomatids with the participation of mosquitoes, we performed a multifaceted study combining the examination of naturally- and experimentally-infected insects using light and electron microscopy and molecular identification of parasites. Our examination of overwintering mosquitoes (genera *Culex* and *Culiseta*) revealed that their guts contained living trypanosomatids, which can be spread during the next season. Experimental infections with *Crithidia* spp. demonstrated that imagines represent permissive hosts, while larvae are resistant to these parasites. We argue that for the parasites with wide specificity, mosquitoes act as facultative hosts. Other trypanosomatids may have specific adaptations for vertical transmission in these insects at the expense of their potential to infect a wider range of hosts and, consequently, abundance in nature.

## 1. Introduction

Trypanosomatids belong to one of the most intensively studied groups of parasitic protists, which is justified by the presence among them of dangerous human pathogens causing vector-borne diseases such as sleeping sickness, Chagas disease, and various leishmaniases [1]. This also determines the scientific interest in monoxenous (parasitizing-only insects) members of the family Trypanosomatidae, which demonstrate large abundance and diversity in nature [2]. While there are many articles focusing on the taxonomy and distribution of these parasites, studies on their biology are still scarce [3]. In particular, trypanosomatid parasites of the ubiquitous mosquitoes have not received much attention thus far.

As potential vectors of malaria and trypanosomes, mosquitoes were among the first insects massively examined for the presence of parasites. Thus, the first species of monoxenous trypanosomatids were characterized in them in as early as the first decade of the 20th century [4,5,6]. Despite numerous records from a wide range of mosquito taxa, during the pre-molecular epoch, only nine nominal species of these flagellates were described, of which two were recognized as synonyms of *Crithidia fasciculata* [7]. The priority of morphology in the systematics of trypanosomatids at that time determined an excessive taxonomic parsimony so that similar-looking flagellates from different hosts were lumped together even if they showed differences in metabolism [8]. As a result, *C. fasciculata* and *Blastocrithidia culicis* were the only repeatedly recorded species [7]. Owing to the availability of laboratory cultures, these two species (especially the first one) became model objects in the studies of trypanosomatid biology [9].

The question of whether mosquitoes can share their flagellates with other insects has been difficult to answer using traditional approaches. However, there have been attempts to assess this using experimental infection. For example, the bedbugs *Cimex lectularius* were shown to be infected when feeding on a culture of *Herpetomonas culicidarum* (synonym of *C. fasciculata*) and produced feces with viable parasites [10]. The same effect with a significant longevity of the infection was observed upon an intrarectal injection of this flagellate to large milkweed bugs *Oncopeltus fasciatus* [11]. The main impact of such findings consisted in doubts about the correctness of classifying trypanosomatids according to their hosts [12,13].

The advent of molecular phylogenetic methods brought significant changes to the knowledge of trypanosomatids in general and to those of mosquitoes in particular. Thus, *Blastocrithidia culicis* was transferred to the genus *Strigomonas*. Although morphologically dissimilar to other members of this taxon, this flagellate proved to share some ultrastructural features with them and bear bacterial endosymbionts of the same origin [14]. Another important advancement was the description of *Paratrypanosoma confusum*, a monoxenous flagellate representing the earliest diverging lineage among trypanosomatids [15]. Importantly, the morphology of this organism as observed with a light microscope does not allow for its discrimination from members of some other monoxenous genera. Therefore, at least some of the old records might include this parasite or related ones identified as *Crithidia, Leptomonas*, or *Herpetomonas* sp. [7].

The analysis of the 18S rRNA sequence of the laboratory culture of *Crithidia luciliae* isolated from the green bottle fly revealed its 100% identity with that of *Crithidia fasciculata* suggesting that this is a single species occurring in both mosquitoes and flies [16]. The use of molecular methods for the analysis of natural infections demonstrated that the diversity of monoxenous trypanosomatids in mosquitoes has been underestimated. This started from the discovery of the abovementioned *Paratrypanosoma* lineage during the survey of West Nile virus and trypanosomes in *Culex* spp. from the USA [17]. Recent studies in Europe directly focused on trypanosomatids in mosquitoes recorded not only *P. confusum* and *Crithidia fasciculata*, but also an undescribed *Herpetomonas* sp., as well as *C. dedva*, *C. brevicula*, and *C. pragensis* [18,19,20]—flagellates previously known from other insect groups such as true bugs, flies, and/or mecopterans [21,22,23]. Thus, mosquitoes apparently share at least some monoxenous trypanosomatids with other hosts. This poses questions about the role of mosquitoes in the circulation of flagellates, the modes of transmission, and the overall relationships in these parasitic systems.

Here we performed a multifaceted study aimed at answering these questions. We analyzed the presence of trypanosomatids in mosquitoes during the summer season and overwintering, performed experimental infections of imagines and larvae of mosquitoes with laboratory cultures of different trypanosomatid species, and investigated the parasites’ development using light and electron microscopy.

## 2. Materials and Methods

### 2.1. Insects Collection and Dissection

**Figure 1:**
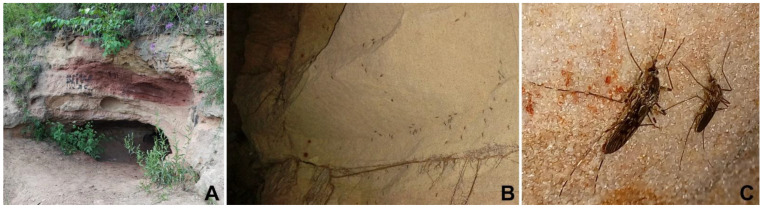
Mosquitoes in the Sablino sandy caves. (**A**)— main entrance to the Zhemchuzhnaya cave (in summer); (**B**)—overwintering mosquitoes on a cave wall; (**C**)—*Culiseta annulata* (left) and *Culex* sp. (right) sitting on a sandy wall next to each other.

Adult overwintering mosquitoes (females) were manually collected from the walls of two Sablino caves (Tosnensky District, Leningrad Region, Russia): Zhemchuzhnaya (59°40′06″ N, 30°48′00″ E) and Santa-Maria (59°39′41″ N, 30°48′02″ E) in January, March and April 2022 (Figure 1, Table S1). Until dissection, the insects were preserved in plastic bottles (~20 insects per each) with a wet cotton disc in a fridge (4–8 °C). The dissection, analysis of infection, and conservation of infected intestine fragments for subsequent DNA isolation were performed as described earlier [24]. The material from positive specimens was also used to establish laboratory cultures, which were cultivated, purified, and cryopreserved as previously described [25].

For the analysis of natural trypanosomatid prevalence in larvae, they were collected in various temporary water bodies in Oksochi village, Novgorod Region (58°39′ N, 32°47′ E), from late April to August 2021. The presence of trypanosomatids was estimated by microscopical examination following dissection or by PCR using the 1127F/1958R primers (see below) in pools consisting of 3–10 individuals.

Human-attacking female mosquitoes were collected in the late summer and early autumn of 2021 in Novgorod, Pskov and Leningrad Regions (Table S2). The captured insects were preserved in individual Eppendorf tubes before dissection for no longer than 24 h. The presence of infection was assessed microscopically. The identification of trypanosomatids was performed by sequencing the 18S rRNA gene (see below) from the infected gut or cultures established from them.

### 2.2. Identification of Insects

The identification of mosquito imagines (*Aedes* spp., *Culiseta annulata*) and differentiation of *Culex torrentium/pipiens pipiens* from other *Culex* spp. were performed using a morphological key [26]. *Culex p. pipiens* and *C. torrentium* females were divided based on the r_2/3_/r_3_ index, calculated from the lengths of the corresponding radial veins of the wings as described earlier [27,28]. The identification of third instar larvae for experimental infections was performed morphologically using the same manual as for imagines [26].

### 2.3. DNA Isolation, PCR, and Sequencing

Total genomic DNA was isolated with the GeneJET Genomic DNA Purification Kit (ThermoFisher Scientific, Waltham, MA, USA) following the manufacturer’s protocol. Specific amplification of a nearly full-length or ~850 bp-long or nearly full-length trypanosomatid 18S rRNA gene was performed using the primer pairs 1127F/1958R [29] and S762/S763 [30], respectively. The PCR fragments were sequenced either using amplification primers, or, in the case of the full-length 18S rRNA gene, with a set of internal primers as described before [31]. In addition to the material collected in this study, the same steps were taken for the analysis of the 18S rRNA gene of the isolate CL6, preserved in the culture collection of parasitic protists at the Zoological Institute RAS [32]. The obtained sequences were deposited in GenBank under the accession numbers OP748970-OP748981.

### 2.4. Phylogenetic Analysis

The nucleotide sequences obtained in this work, along with the related ones retrieved from GenBank, were aligned by MAFFT v. 7.490 [33] using the E-INS-I algorithm. The alignment was trimmed using trimAl v. 1.2 [34] with the 50% gap threshold, giving the resulting matrix of 73 taxa with 2140 nucleotide positions. A maximum likelihood tree was inferred in IQ-TREE v. 2.2.0 [35] under the TIM3e + I + G4 substitution model automatically selected by the ModelFinder module [36]. Edge support was estimated using the standard bootstrap method with 1000 replicates. The phylogenetic reconstruction was also performed using MrBayes v. 3.2.7 [37] run under the GTR + I + G model for 4,000,000 generations with every 100th of them sampled. Other parameters were set by default.

### 2.5. Experimental Infections

A series of experiments with the infection of mosquito larvae and imagines was performed using laboratory cultures of the following trypanosomatids: *Crithidia fasciculata* isolate Cf-C1 [38], *C. brevicula* Nbr [39], as well as *C. dobrovolskii* M1173 and *C. brevicula* M1183 isolated in this work.

#### 2.5.1. Larvae

Fallen leaves were collected from under the snow at the edge of a pond, where large quantities of *Aedes cantans* larvae were spotted in the last year. These leaves, containing mosquito eggs, were placed in a bucket with cold (+4 °C) tap water filtered with a household filter. After 2 days of incubation (the temperature gradually increased to +18°C), the water with the hatched larvae was transferred to a new bucket, while the leaves were removed. Microorganisms developing in this water served as food for the growing larvae.

In other cases (*Aedes riparius*, *A. communis*, *A. punctor*, and *Culex torrentium*), the larvae were taken directly from four natural temporary water bodies in the village of Oksochi in the Novgorod Region from late April to July. For the confirmation of their uninfected status, 50% of the randomly selected individuals were dissected before the experiments.

Experimental infections were performed using third instar larvae by placing them in filtered water containing a trypanosomatid culture with the final concentration of 1 × 10^4^ cells/mL. After one hour, the larvae were washed in three changes of filtered water (with 5 min incubation in each) and finally transferred to the water, where they had been grown before. Dissections were performed only for the larvae that remained alive.

#### 2.5.2. Imagines

Mosquito larvae and/or pupae collected in nature were bred in water infused with dry leaves/grass water infusion until metamorphosis. The emerging imagines were maintained in transparent plastic bottles with pieces of cotton soaked in water and/or sugar syrup as described previously [40]. For infection, these liquids were replaced for 10–12 h with a 4-day-old trypanosomatid culture. The infected insects were dissected after defined time intervals (1, 2 and 4 days). The absence of prior infection was assessed by dissecting eighteen control individuals.

## 3. Results

### 3.1. Natural Trypanosomatid Infections in Overwintering Mosquitoes

In one of the two sampled caves, Santa-Maria, trypanosomatids were not detected in any of the 74 examined mosquitoes (Table 1). In the Zhemchuzhnaya cave, trypanosomatids were revealed both in January and in April, with the overall prevalence lower at the later time point due to the remarkably lower proportion of infected *Culex p. pipiens*. However, these differences were not statistically significant according to the “N-1” Chi-square test (*p* = 0.2968 for the overall prevalence and *p* = 0.1916 for that in *Cx. p. pipiens*) [41]. Trypanosomatids were found in all three species collected: *Cx. p. pipiens*, *Cx. torrentium* and *Culiseta annulata.*

**Figure 2:**
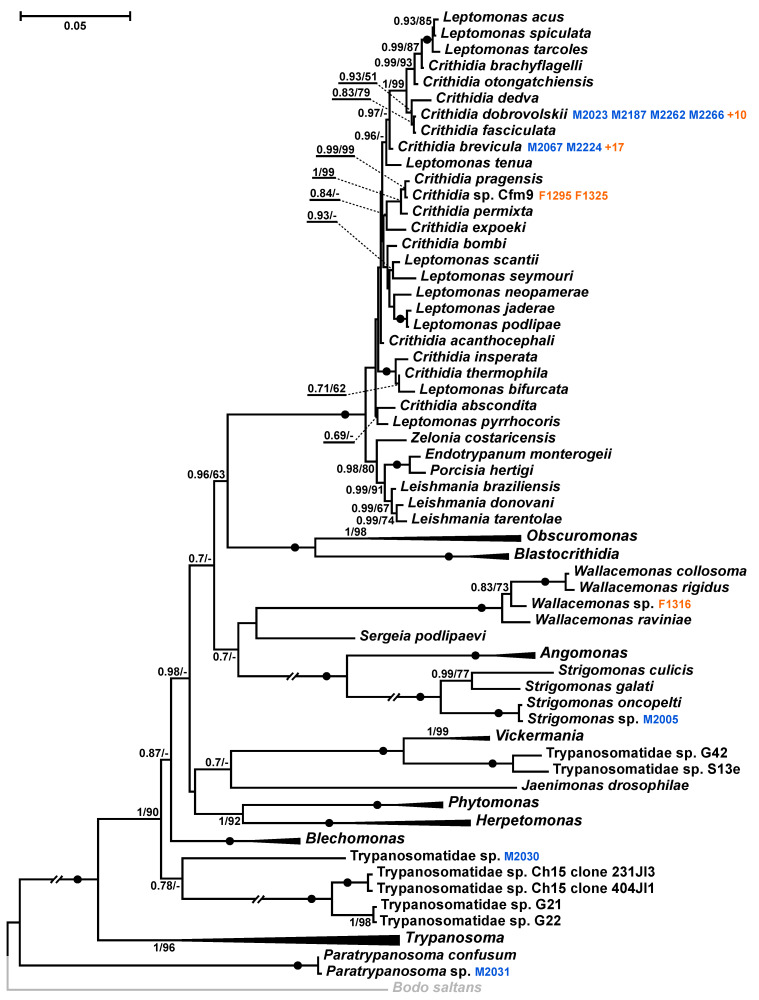
Maximum likelihood phylogenetic tree based on 18S rRNA gene sequences. Isolates from overwintering mosquitoes are in blue, those from the end of the warm season are in orange (only the total number is shown if it exceeds four). The clades of genera not containing such isolates are collapsed. Posterior probabilities and bootstrap values from maximum likelihood analysis are shown at the nodes (values below 0.5 or 50% are replaced with dashes or omitted). Dots mark branches with maximal support by both methods. The tree was rooted with the sequence of *Bodo saltans* (Eubodonida) as an outgroup (shown in grey). Double-crossed branches are shortened by 50 %. The scale bar denotes the number of substitutions per site.

The analysis of the 18S rRNA gene sequences from the positive specimens demonstrated that the parasites belonged to five trypanosomatid species. Of these, two, namely *Crithidia dobrovolskii* and *C. brevicula*, were detected four (in all three host species) and two times (on both *Culex* spp.), respectively. The remaining three specimens (all from *Cx. p. pipiens*) contained unique species of flagellates: M2005 was ~99.8% identical to *Strigomonas oncopelti,* M2031 was ~99.8% identical to *Paratrypanosoma confusum*, and M2030 proved to be a representative of a new trypanosomatid lineage not recorded before (Table S1, Figure 2). None of these three species could be revealed in the cultures established from the corresponding specimens. *Crithidia dobrovolskii* and *C. brevicula* were detected there instead (Table S1), suggesting mixed infections, in which they represented minor components, a situation quite common in insects [42,43,44,45,46,47].

Given that *C. dobrovolskii* is very closely related to *C. fasciculata* and information on its distribution is rather scarce, because this species was described quite recently [48], we decided to check the phylogenetic position of one culture, CL6, preserved in the culture collection of parasitic protists at the Zoological Institute RAS [32]. This culture was isolated in the same geographic region as *C. dobrovolskii* (i.e., the Northwestern Federal District) and, likewise, was very close to *C. fasciculata*, but by isoenzyme profile [49]. The 18S rRNA gene sequence obtained from the culture CL6 was 100% identical to that of *C. dobrovolskii.* Thus, the damsel bug *Nabis limbatus*, from which it has been isolated, can be added to the list of recorded hosts.

### 3.2. Natural Trypanosomatid Prevalence in Adult Mosquitoes at the End of the Warm Season

To understand the situation with trypanosomatid infections at the end of the warm season, mosquitoes (all proven to be representatives of the genus *Aedes*) were analyzed in three regions of the Northwestern Federal District (Table S2). The prevalence of all trypanosomatids was quite high, ranging from 64 to 94%, and the overwhelming majority of these infections were due to monoxenous species (Table 2 and Table S2). Although we could detect only a single dixenous parasite species, a member of the TthII clade of *Trypanosoma theileri* complex [24], the monoxenous ones were predominantly represented by *C. brevicula* and *C. dobrovolskii* along with less frequent *Crithidia* sp. Cfm9 (closely related to *C. pragensis*) and *Wallacemonas* sp. (Table S2, Figure 2).

### 3.3. Natural Trypanosomatid Prevalence in Mosquitoe Larvae

Considering the high prevalence of trypanosomatids in adult mosquitoes, it was decided to assess whether the infections are present in larvae. In total, 371 larvae belonging to different species of the genera *Aedes* and *Culex* sampled from late spring to late summer were examined, but trypanosomatids could not be detected in any of them (Table 3).

### 3.4. Experimental Infections

In addition to the assessment of the natural trypanosomatid prevalence in larvae, a series of experiments on their infection was performed using laboratory cultures of three trypanosomatids: *Crithidia fasciculata*, the canonical parasite of mosquitoes, as well as the two most common species according to our findings—*C. brevicula* and *C. dobrovolskii*. In all our experiments, trypanosomatids could be observed in the intestine of all or the majority of larvae 2 h after exposure to cultures. However, the parasites were unable to establish a solid infection, and, after 24 h, purging was evident in all cases except for *C. brevicula* in *Aedes cantans* (Table 4). Even in that experiment, the parasites could not be detected after four days.

The results were quite different for the imagines, the infection of which was attempted using the cultures of *C. brevicula* and *C. dobrovolskii* isolated in this study (M1183 and M1173, respectively). A high proportion of insects were infected even after four days with no signs of decline in prevalence (Table 5), while the intensity of the infections increased (see below). Given that *C. brevicula* is a species previously documented in a wide range of hosts [19,21,50], we tested whether a culture from a phylogenetically distant and physiologically distinct insect, the predatory damsel bug *Nabis brevis*, can be infective for mosquitoes. Despite over 30 years of continuous cultivation, the culture Nbr demonstrated the ability for a stable infection in *Aedes communis* (Table 5).

### 3.5. Trypanosomatid Development in the Gut

The success with the experimental infection of imagines suggested a more detailed investigation of parasites’ development in the gut to make further conclusions on the competence of mosquitoes as hosts of trypanosomatids.

**Figure 3:**
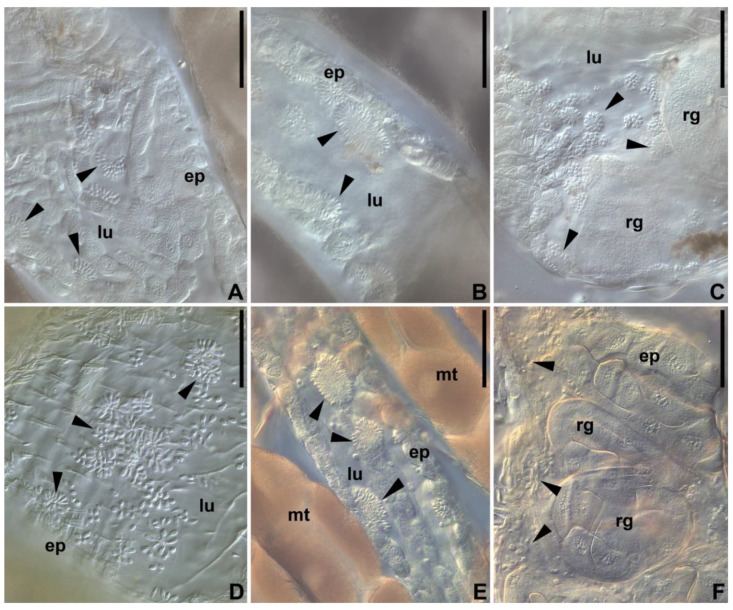
Experimental infections of mosquitoes with two trypanosomatid species (DIC). (**A**–**C**)—*Crithidia brevicula*; (**D**–**F**)—*Crithidia dobrovolskii*. (**A**,**D**)—pylorus; (**B**–**E**)–ileum; (**C**–**F**)—rectal ampulla. ep—gut epithelium; lu—gut lumen; mt—Malpighian tubules; rg—rectal glands. Arrows point to trypanosomatid aggregates (rosettes). Scale bars—60 µm.

Both *C. brevicula* and *C. dobrovolskii* developed similarly from the beginning of the infection. After 24 h, most flagellates were revealed in the pylorus and ileum, where few-celled rosettes started to appear. Two days post infection, the rosettes were observed along the entire length of the hindgut, i.e., from the pylorus to the rectum (Figure 3). Development was not synchronous in different host individuals. While in some of them the parasites covered the hindgut cuticle with a carpet of attached cells 2–4 days post infection, in others only rare rosettes and/or a few swimming flagellates were observed at that time.

**Figure 4:**
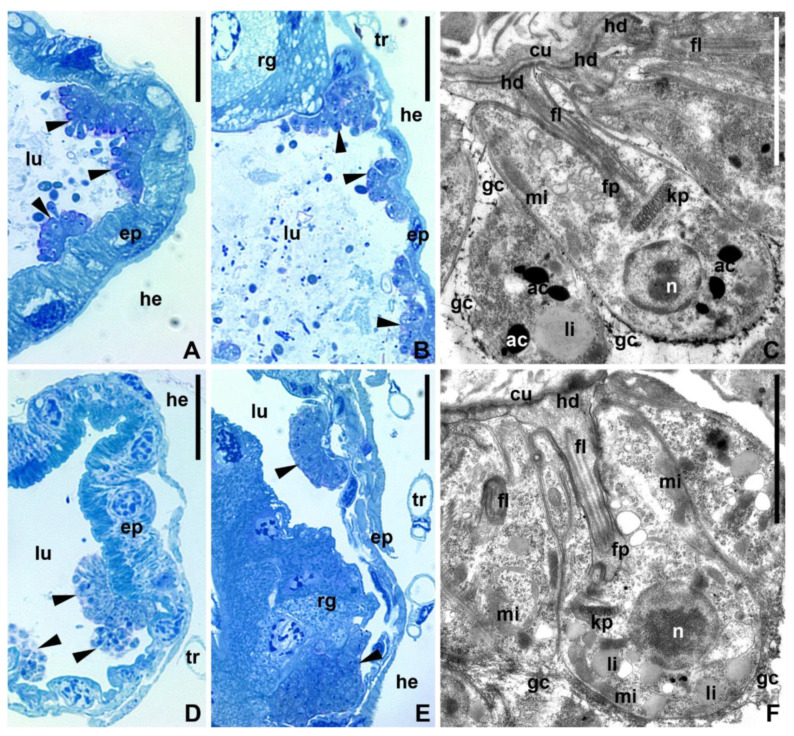
Trypanosomatids in the hindgut of experimentally infected mosquitoes (96 h). (**A,B**,**D**,**E**)—semithin sections stained with methylene blue-azure stain (light microscopy); (**C**–**F**)—TEM. (**A**–**C**)—*C. brevicula*; (**D**–**F**)—*C. dobrovolskii*; (**A**,**C**,**D**,**F**)—ileum, (**B**–**E**)—rectum. Ac—acidocalcisome; cu—cuticle; ep—epithelium fl—flagellum; fp—flagellar pocket; gc—glycocalyx; hd—hemidesmosome; he—hemocoel; kp—kinetoplast; li—lipid droplets; lu—intestinal lumen; mi—mitochondrion; n—nucleus; rg—rectal gland; tr—trachea; arrowheads—attached parasites. Scale bars: (**A**,**B**,**D**,**E**)—20 µm; (**C**–**F**)—2 µm.

The development in the pylorus and ileum was similar in both species: the proliferation of cells within a rosette led to its expansion as a monolayer carpet enclosed into a unite matrix (Figure 4A,D). In the mosquito rectum, the cells of both *Crithidia* spp. also attached to the cuticular lining in large numbers. They settled not only on the wall of the rectal ampulla, but also on the surface of the rectal glands (Figure 4B,E).

The analysis of the ultrastructure showed that both species of parasites harbored a thick (~0.5–0.7 µm) layer of fibrillar glycocalyx (Figure 4C,F). The glycocalyces of neighboring cells fused together, forming a unite matrix that enveloped the whole rosette/cluster. The fixation of cells to the hindgut cuticle was achieved using a widened tip of a shortened flagellum, which formed a zonal hemidesmosome at the inner surface of its membrane (Figure 4C,F). The intraflagellar matrix in the tip was filled with bundles of filaments. The nucleus, kinetoplast, mitochondrial branches, and lipid droplets were observed in the cytoplasm of the attached flagellates of both species. However, acidocalcisomes were present only in *C. brevicula*, but not in *C. dobrovolskii*, which instead demonstrated a more developed contractile vacuole complex (Figure 4C,F).

## 4. Discussion

Monoxenous trypanosomatid infections in mosquitoes deserve attention for several reasons. Firstly, there is a growing evidence that these flagellates under certain conditions can cause infections in humans, dogs, and other vertebrates [2]. Mosquitoes and other bloodsucking insects, as compared to their kin with other feeding habits, should have higher chances to deliver their parasites to a vertebrate body. Secondly, the occasional infections of vertebrates at a long evolutionary distance apparently led to the origin of dixenous parasite lineages [51] and mosquitoes might be a group that facilitated the exploration of vertebrate hosts. Thirdly, the most divergent trypanosomatid known to date, *Paratrypanosoma confusum*, inhabits mosquitoes [15]. Thus, these insects might be among the earliest hosts of Trypanosomatidae. Finally, the abundance of mosquitoes suggests that they may also play an important role in the circulation of monoxenous trypanosomatids in ecosystems.

Previous studies have already shown that mosquitoes can be naturally infected not only by trypanosomes, but also by various monoxenous trypanosomatid species [18,19,20]. The most important in this respect is the large-scale screening in Austria, which documented the presence of monoxenous trypanosomatids in various genera and species of mosquitoes [19]. Interestingly, our analysis of infections at the end of the warm season showed a very similar composition of parasite species. Although formally only a single species, namely *Crithidia brevicula*, is shared between the two studies, the 18S rRNA gene fragment sequenced by Austrian researchers does not allow discrimination of *C. fasciculata* and *C. dobrovolskii*, as well as *C. pragensis* and *Crithidia* sp. Cfm9. Therefore, it is very likely that all *Crithidia* spp. in both cases are the same. Then, the main differences consist in the unique infections by *Wallacemonas* sp. in our dataset and *Herpetomonas* sp. (from the mosquito species *Coquillettidia richardii*, which we did not examine) in the Austrian dataset [19].

The uninfected status of larvae from natural habitats, where they theoretically could massively acquire trypanosomatids given their filtration feeding habit [52] and the failure to establish such an infection in experimental conditions suggest that this developmental stage is resistant to the parasites investigated here. This agrees with the observation that trypanosomatid infections in insect larvae are an exception rather than a rule [3]. However, this contrasts with previously published successful results of the transmission of *Crithidia fasciculata* (one of the species that we used in our experiments) from imagines to larvae, which then preserved the parasites even after metamorphosis [53]. This contradiction is not surprising given that the actual identity of the flagellate used in that work is obscure: the authors have neither isolated a culture nor presented an image of the studied parasite. At that time, all trypanosomatids without an undulating membrane found in mosquitoes were assigned to *C. fasciculata* [8], but nowadays it is clear that this could easily be a member of a different genus. Of note, experiments performed by Franklin G. Wallace with a culture of *Crithidia fasciculata* have shown results in line with ours [54]. In any case, as judged by some publications that are over a century old, it appears that certain monoxenous trypanosomatids can infect mosquitoes at the larval stage and undergo transphasic transmission to imagines [4,55,56]. The available images allow it to be stated that these parasites are morphologically distinct from *C. fasciculata*, so their phylogenetic position is to be determined.

Although trypanosomatids infecting mosquitoes at the larval stage apparently exist, they seem to be rare. Thus, the high (up to 88%) prevalence of monoxenous trypanosomatids that we revealed in *Aedes* spp. can be explained by the gradual accumulation of parasites in mosquito populations during the warm season, at the end of which we analyzed them. To fulfil the energy needs, both male and female mosquito imagines feed on sugar-containing liquids, such as honeydew, nectar, fruit exudates, or sap [17,52]. Contamination of these liquids (or water, which they also drink) with feces of trypanosomatid-infected insects can serve as a source of infection. It is then understandable why these mosquitoes contained parasites with a wide host range. *Crithidia brevicula* has been considered a specific parasite of *Nabis brevis*, a predatory damsel bug*,* after which it received its name. The specificity of the relationships was justified by the presence of the parasite in overwintering bugs [39]. Later, the same trypanosomatid species was detected in other true bugs (predatory Gerridae (water striders) and phytophagous Miridae (meadow bugs)), mosquitoes, as well as flies of five different families (Calliphoridae, Muscidae, Heleomyzidae, Sepsidae, and Antomyidae) [19,21,50]. Our experimental infections demonstrated that this species progressively develops in *Aedes* ensuring stable infection, which can also be considered specific. Moreover, we revealed that the type culture of *C. brevicula* (Nbr), which has been isolated from *Nabis brevis*, also effectively infects these mosquitoes. Finally, this trypanosomatid was also detected in overwintering *Culex* spp. All these facts confirm that *Crithidia brevicula* is a generalist species.

*Crithidia dobrovolskii* has been described recently and data on its distribution over host taxa are still scarce. Beyond mosquitoes, it has been previously recorded from only two fly species: parasitoid *Lypha dubia* (Tachinidae) and the tsetse *Glossina fuscipes* (Glossinidae) [48,57]. Owing to the close relatedness of this species to *C. fasciculata*, we extended the list of recorded hosts by adding the damsel bug *Nabis limbatus*, from which the culture CL6 (showing the same affinity by isoenzyme profiles) had been isolated almost 40 years ago, but has not yet been analyzed by 18S rRNA gene sequencing [32,49].

The third species of the genus *Crithidia* that we documented in mosquitoes is an undescribed one, closely related to *C. pragensis*. In addition to mosquitoes, it has also been isolated (as the culture Cfm9) from a damsel bug, namely *Nabis flavomarginatus* [49]. The occurrence of this species is relatively low both in our material and (if we assume that this was the same species, see above) in the Austrian large-scale study [19]. In the absence of additional data, it is not possible to judge whether this is due to the non-specificity of this trypanosomatid to mosquitoes or its overall lower abundance in the studied region.

It is notable that all three *Crithidia* spp. documented in this work have been previously found in damsel bugs. *Crithidia dedva* is one more species of that kind, since it has been originally described from *Nabis flavomarginatus* [22] and later recorded in *Culex* sp. [18]. Damsel bugs are known as very abundant predators feeding on a wide range of insects [58], including mosquitoes [59]. The fact that certain trypanosomatid species with proven or presumed wide specificity are shared between *Nabis* spp. and mosquitoes points to these parasites’ circulation between the two kinds of hosts. Both mosquitoes and damsel bugs, which also feed on plant juices [58], can be infected via fecal contamination of the substrate, but the latter also have an opportunity to acquire parasites by predation on the former. We propose that owing to the high abundance and motility, mosquitoes ensure effective growth and spreading of trypanosomatid populations during the warm season. *Aedes* spp. overwintering at the egg stage are not able to support the continuity of the parasites’ circulation in ecosystems and therefore represent the so-called facultative hosts. Conversely, their relatives of the genera *Culex* and *Culiseta*, which outlive wintertime as imagines, preserve their parasites to the next year. However, our experiments demonstrated the inability of *Crithidia* spp. to infect mosquito larvae, suggesting that these trypanosomatids cannot be transmitted from overwintered females (they die soon after laying eggs [52]) to their progeny. The resting stages of *Crithidia* spp., the endomastigotes, preserve viability for about 10 days [3], which is not enough for the emergence of new imagines in the spring [52]. Therefore, without participation of other hosts (e.g., damsel bugs), the circulation of such parasites would be broken.

The nature of the single infection by *Wallacemonas* sp. detected in *A. diantaeus* is unclear. Members of this genus were encountered in various flies and predatory bugs [21,31,60,61,62]. Recently, *W. raviniae*, which had been originally described from a flesh fly *Ravinia* sp. (Sarcophagidae), was found to develop in a horsefly in co-infection with a more common *Trypanosoma theileri*-like trypanosome [43]. Given the similarity to *Crithidia* spp. in morphology and localization (in the hindgut), it is tempting to also extrapolate the wide specificity and overwintering in a suitable host. However, the transient nature of such infections cannot be excluded.

The situation with other monoxenous trypanosomatids encountered in mosquitoes appears to be different. The presence of three non-*Crithidia* species in overwintering female mosquitoes suggests the specificity of these infections. The observed low prevalence cannot be considered a counterargument in this respect, since it is explainable from the point of view of mosquito biology. Mosquitoes that overwinter as imagines emerge from pupae shortly before going to caves and have a restricted time to feed and acquire parasites [52]. Of note, they rarely feed on blood in this period, and, consistently, we detected no trypanosomes in their intestines.

The specificity of these three species may be different. All records of *Paratrypanosoma* available in the literature are from *Culex* spp.: the only described species, *P. confusum*, or closely related flagellates have been detected in *Cx. pipiens*, *Cx. tarsalis*, and *Cx. fuscocephala* [15,17,20,63]. This points to a narrow specificity of paratrypanosomes (at species or genus level) and suggests that there should be an adaptation ensuring continuous circulation of these parasites in nature, such as the ability to infect larvae.

To the best of our knowledge, *Strigomonas oncopelti* has not been documented in any insects after its original description (under the name *Herpetomonas oncopelti* using five isolates from different sources: seed bugs *Oncopeltus* sp., *O. fasciatus,* and *Lygaeus kalmii* (all Lygaeidae), as well as milkweeds *Asclepias syriaca* and *A. nivea* (Apocyanaceae) [64]. That description was based on mixed material and some cells were later recognized as a different species described as *Leptomonas oncopelti* [65]. Although at first these cultures required different media to be established, later they became indistinguishable by serological and biochemical criteria [66], suggesting that at least in some of them species replacement might occur. Only one out of five cultures isolated upon description was widely studied and deposited into the American Type Culture Collection. However, its actual origin is now considered to be “unknown”, and justified doubts have been cast on its ability to inhabit any of the hosts listed in its description [8]. Indeed, there were no more records of morphologically similar trypanosomatids from any plants [67], nor phylogenetically related ones in any bugs of the family Lygaeidae sampled throughout the world [42,45,46,60,68,69,70,71]. Interestingly, the paper describing *Strigomonas oncopelti* also contained a description of *Herpetomonas culicidarum* based on two cultures isolated from the mosquitoes *Culex pipiens* and *Anopheles quadrimaculatus* [64]. Later it was demonstrated that these two cultures were distinct: the one from *Cx. pipiens* did not require hemin for its growth —a feature now unambiguously indicating the presence of bacterial symbionts, which is characteristic of, e.g., *Strigomonas* spp. [1]. However, later both mosquito-derived isolates were included into *Crithidia fasciculata* as varieties [8] and became forgotten. The abovementioned information and the fact that *Strigomonas culicis* is a parasite of mosquitoes suggest that the same can be true for *S. oncopelti* and its close relative detected in our work.

Little can be said about the third non-*Crithidia* species from overwintering mosquitoes. It belongs to a mysterious group, which has not been sampled before, at least in modern time, when molecular methods started to be used. As in the case of *S*. cf. *oncopelti*, this demonstrates how much the trypanosomatids of mosquitoes are still underexplored. However, for this particular species, in addition to the presence in an overwintering mosquito and the absence of records from other hosts, there is one more argument for the specificity of this parasite: it was revealed in Malpighian tubules, which represent a very peculiar localization, in contrast to the more common midgut and hindgut [3]. Importantly, in *Blastocrithidia papi*, this localization is associated with overwintering and is necessary for the coordination of the host and parasite life cycles so that the massive production of infective cyst-like amastigotes is timed to oviposition in females [72]. It would be interesting to investigate whether the same occurs with this obscure mosquito trypanosomatid.

## 5. Conclusions

The mosquitoes studied in this work harbored various trypanosomatids showing different life strategies. *Crithidia brevicula*, *C. dobrovolskii*, and, likely, other mosquito-inhabiting *Crithidia* spp. represent parasites with a wide host range. They are able to parasitize not only different kinds of mosquitoes, but also other insects, such as flies and true bugs. Infecting mosquitoes, which are highly abundant and motile insects, ensures efficient growth of trypanosomatid populations and dissemination of infective forms in the ecosystems. However, since these species are unable to infect mosquito larvae, the continuity of these parasites’ circulation apparently requires involvement of other hosts, most likely the predatory damsel bugs. Thus, for these species, mosquitoes represent facultative hosts.

Trypanosomatids such as *Paratrypanosoma* cf. *confusum*, *Strigomonas* cf. *oncopelti*, and a representative of a new previously undocumented lineage were found in overwintering *Culex p. pipiens* suggesting their narrow specificity and, consequently, obligate status of the host in such relationships. We propose that in the absence of long-lived resting stages, which were not observed in any trypanosomatids from mosquitoes, the circulation of such species requires infection in larvae with subsequent transphasic transmission to imagines.

## Figures and Tables

**Table 1:** Prevalence of trypanosomatid infections in overwintering mosquitoes.

Collection Date	Cave	*Culex p. pipiens*	*Culex torrentium*	*Culiseta annulata*	Overall
26.01.22	Zhemchuzhnaya	3/55 (5.5%)	1/22 (4.5%)	1/18 (5.6%)	5/95 (5.3%)
11.03.22	Santa-Maria	0/32 (0%)	0/40 (0%)	0/2 (0%)	0/74 (0%)
13.04.22	Zhemchuzhnaya	2/111 (1.8%)	2/37 (5.4%)	0/0	4/148 (2.7%)

**Table 2:** Trypanosomatid prevalence in *Aedes* spp. captured in late summer–early autumn.

Location	Prevalence	
	Total	Monoxenous ^1^
Novgorod Region	14/19 (74%)	13/19 (68%) or 10/19 (53%) ^2^
Pskov Region	7/11 (64%)	6/11 (55%)
Leningrad Region	16/17 (94%)	15/17 (88%)

^1^ Minimal value deduced from all available data. ^2^ Values estimated from both sequence and morphology data, or only from sequence data, respectively.

**Table 3:** Analysis of natural trypanosomatid prevalence in mosquito larvae.

Species	Month	Assessment	Prevalence
*Aedes riparius*	Late April	Microscopy or PCR	0/41
*A. punctor*	May	Microscopy or PCR	0/54
*A. communis*	May	Microscopy or PCR	0/53
*Culex p. pipiens*	August	Microscopy	0/13
*Cx. torrentium*	July–August	Microscopy or PCR	0/210

**Table 4:** Prevalence of trypanosomatids in experimental infections of larvae.

Host	Parasite	Control	2 h	24 h	4 d	Pupae	Imagines	Died ^3^
*Aedes cantans* ^1^	*Crithidia brevicula* M1183	0/3	8/11	4/7	0/9	-	-	0
*Aedes punctor* ^2^	*Crithidia fasciculata*	0/58	10/10	0/15	-	0/17	0/9	7
*Aedes communis* ^2^	*Crithidia fasciculata*	0/30	7/7	0/7	-	0/10	0/6	0
*Culex torrentium* ^2^	*Crithidia fasciculata*	0/70	10/10	0/30	-	0/20	0/8	2
*Culex torrentium* ^2^	*Crithidia dobrovolskii*	0/60	10/10	0/30	-	0/14	0/5	1

^1^ Bred in the lab, therefore the number of control specimens is limited. ^2^ Collected in nature, the control specimens for these experiments are also included in the statistics of the Table 1. **^3^** Insects that were found dead at any stage of the experiment were excluded from the analysis. Dashes mean “not assessed”.

**Table 5:** Trypanosomatid prevalence in experimental infections of *Aedes communis* imagines.

Parasite	1 d	2 d	4 d	Control
*Crithidia dobrovolskii*	4/9 (44%)	3/4 (75%)	5/5 (100%)	0/18 (0%)
*Crithidia brevicula* M1183	2/3 (67%)	18/38 (47%)	16/29 (55%)	
*Crithidia brevicula* Nbr	-	-	2/2 (100%)	

Dashes mean “not assessed”.

## Data Availability

The sequences obtained in this study were submitted to GenBank and are available under accession numbers OP748970-OP748981.

## Supplementary Materials

The following supporting information can be downloaded at: https://www.mdpi.com/article/10.3390/pathogens11111326/s1, Table S1: Analysis of trypanosomatid presence in overwintering mosquitoes; Table S2: Analysis of trypanosomatid presence in mosquitoes captured in the late summer or early autumn.
